# Retraction: The application of key feature extraction algorithm based on Gabor wavelet transformation in the diagnosis of lumbar intervertebral disc degenerative changes

**DOI:** 10.1371/journal.pone.0292208

**Published:** 2023-09-25

**Authors:** The PLOS ONE Editors

The *PLOS ONE* Editors retract this article [1] because it was identified as one of a series of submissions for which we have concerns about peer review integrity and similarities across articles. These concerns call into question the validity and provenance of the reported results. We regret that the issues were not identified prior to the article’s publication.

TY, NL, JL, QH, YL, and HZ did not agree with the retraction. RL, YY, WC, and QW either did not respond directly or could not be reached.
